# Development of a Detailed Chemical Kinetic Model for 1-Methylnaphthalene

**DOI:** 10.3390/molecules29235660

**Published:** 2024-11-29

**Authors:** Junjie Liang, Qianlong Zhang, Yijun Heng, Gesheng Li, Ke Yang, Ruiyang Wang, Fan Dong, Neng Zhu

**Affiliations:** 1 School of Naval Architecture, Ocean and Energy Power Engineering, Wuhan University of Technology, Wuhan 430063, China; liangjunwh@whut.edu.cn (J.L.); 286160@whut.edu.cn (Q.Z.); yijunheng@whut.edu.cn (Y.H.); 286119@whut.edu.cn (R.W.); 2 National Engineering Research Center of Ship & Shipping Control System, Shanghai 200135, China; 3 College of Mechanical and Electrical Engineering, Zhejiang Business Technology Institute, Ningbo 315012, China; 4 School of Transportation and Logistics Engineering, Wuhan University of Technology, Wuhan 430063, China; ky013@uark.edu; 5 Department of Industrial Engineering, University of Arkansas, Fayetteville, AR 72701, USA; 6 Dongfeng Motor Corporation Research & Development Institute, Wuhan 430058, China; dongfan@dfmc.com.cn; 7 School of Automotive and Transportation Engineering, Wuhan University of Science and Technology, Wuhan 430081, China

**Keywords:** 1-methylnaphthalene, detailed kinetic model, ignition delay times, chemical kinetic analysis

## Abstract

1-Methylnaphthalene is a critical component for constructing fuel surrogates of diesel and aviation kerosene. However, the reaction pathways of 1-methylnaphthalene included in existing detailed chemical kinetic models vary from each other, leading to discrepancies in the simulation of ignition and oxidation processes. In the present study, reaction classes and pathways involved in the combustion of 1-methylnaphthalene were analyzed, and effects of rate constants of reactions related to 1-methylnaphthalene and its significant intermediates on ignition delay times and species concentration profiles were discussed, involving hydrogen abstraction and substitution reactions of 1-methylnaphthalene, oxidation, isomerization, and addition reactions of 1-naphthylmethyl, hydrogen abstraction and oxidation reactions of indene, as well as the oxidation of indenyl and naphthalene. On this basis, a new detailed chemical kinetic model for 1-methylnaphthalene was developed, which includes 1389 species and 7185 reactions. The validation of this mechanism shows that it can predict accurately the available experimental ignition delay times, species concentration profiles, and laminar flame speeds of 1-methylnaphthalene. Finally, reaction paths and sensitivity analysis of ignition delay times were performed using the proposed reaction mechanism, and the result shows that the conversion of 1-methylnaphthalene to 1-naphthaldehyde plays an important role in its ignition.

## 1. Introduction

Diesel fuel remains the primary fuel to power large electrical equipment and the pilot fuel to ignite other fuels with high octane numbers such as natural gas and ammonia. As diesel is a complex mixture comprising thousands of components, existing studies often formulate fuel surrogates to investigate the combustion characteristics of real diesel [1,2,3,4,5]. Diesel typically contains about 15–40% aromatic hydrocarbons, with polycyclic aromatic hydrocarbons (PAHs) playing a crucial role in soot formation during diesel combustion. As a bicyclic aromatic hydrocarbon, 1-methylnaphthalene is frequently selected as a key component in the formulation of diesel surrogate fuels [6,7]. Additionally, due to its high energy density form, it is also commonly used in the development of aerospace fuel models [8].

Establishing a numerically coupled model of chemical kinetics and fluid dynamics facilitates in-depth research on fuel combustion performance in engines and their emission characteristics. An accurate chemical kinetics model is essential for this research. Currently, researchers have conducted numerous experimental studies and detailed mechanism analyses on the combustion characteristics of 1-methylnaphthalene. The experimental studies primarily involve measuring the ignition delay times of 1-methylnaphthalene using shock tubes (ST) and rapid compression machines (RCM). These experiments are conducted under equivalence ratios ranging from 0.5 to 1.5, pressures of 10 to 40 bar, and temperatures between 800 and 1400 K [9,10,11]. Additionally, the jet-stirred reactor (JSR) has been employed to measure species concentration profiles under equivalence ratios ranging from 0.5 to 1.5, pressures of 1 to 10 atm, and temperatures between 800 and 1400 K [12]. Laminar flame speeds of 1-methylnaphthalene at pressure of 1 bar, temperatures of 425, 445, and 484 K, and equivalence ratios between 0.8 and 1.35 were measured through the outwardly propagating spherical flame method [13].

Regarding the mechanisms of 1-methylnaphthalene, detailed kinetic models have been proposed by Wang et al. [11], Mati et al. [12], and Narayanaswamy et al. [14]. Additionally, a diesel surrogate fuel Creck mechanism developed by Ranzi et al. [15] incorporates the reaction pathways of 1-methylnaphthalene. Nobili et al. [13] updated the 1-methylnaphthalene reaction mechanism on the basis of the Creck kinetic model [14] by applying analogy and rate rules from toluene. Jin et al. [16] also proposed a kinetic model for PAHs, which includes 1-methylnaphthalene. Mati et al. [12] developed a detailed kinetic model that includes 146 species and 1041 reactions, validating species concentration profiles with good agreement between the predicted and experimental values. However, this mechanism only accounts for the consumption pathways of 1-methylnaphthalene to 1-naphthylmethyl (A_2_ĊH_2_) and naphthalene (C_10_H_8_), omitting hydrogen abstraction reactions, substitution reactions involving hydrogen atoms on the ring with radicals, or fuel decomposition reactions. These reactions are important consumption pathways for 1-methylnaphthalene, and along with hydrogen abstraction from the methyl group, they significantly enhance the fuel’s ignition process. Wang et al. [11], building on the kinetic models developed by Bounaceur et al. [17] and Mati et al. [12], incorporated relevant reactions of hydrogen atoms on the 1-methylnaphthalene ring and created a detailed kinetic model comprising 662 species and 3864 reactions. However, this model does not account for some PAHs, such as acenaphthylene, phenanthrene, and pyrene, which could provide additional pathways for the conversion of 1-methylnaphthalene to other PAHs, thereby more accurately reflecting the complexity of real diesel. Moreover, under conditions with an equivalence ratio of 1.5 and a pressure of 10 bar, the simulated ignition delay times exceed the experimental values. Narayanaswamy et al. [14], based on the small-molecule hydrocarbon mechanism by Blanquart et al. [18], developed a detailed kinetic model of 1-methylnaphthalene that includes 158 species and 1804 reactions, incorporating some aromatic species. However, this model also lacks the relevant reactions of hydrogen atoms on the 1-methylnaphthalene ring, and the simulated ignition delay times at high temperatures remain higher than the experimental values. In the diesel surrogate fuel Creck mechanism developed by Ranzi et al. [15], the hydrogen abstraction reactions of hydrogen atoms on the benzene ring of 1-methylnaphthalene are included, but the substitution reactions of radicals with hydrogen atoms are not covered. Based on the Creck mechanism, Nobili et al. [13] introduced reactions related to intermediate products including methylnaphthol radical (${\;\dot{O}C}_{10}H_{6}{\mathrm{CH}}_{3}$), ethyl-naphthalene (C_10_H_7_C_2_H_5_), and naphthoquinone (C_10_H_6_O_2_), and a mechanism for 1-methylnaphthalene was proposed. Through analyzing this mechanism, it can be found it does not take into account the isomers of these intermediate products such as methylnaphthol radical, 1-naphthylmethyl(A_2_ĊH_2_), and naphthoquinone.

Furthermore, certain intermediates in the aforementioned detailed mechanisms remain contentious, particularly the formation and consumption of 1-naphthylmethyl-oxy ($A_{2}{\mathrm{CH}}_{2}\dot{O}$), which lacks consensus. In contrast, the analogous benzyl-oxy ($A_{1}{\mathrm{CH}}_{2}\dot{O}$) plays an important role in toluene combustion. Similarly, the formation of indene-oxy ($C_{9}H_{7}\dot{O}$) and indanone (C_9_H_6_O) affects the conversion of indenyl to benzene derivatives. Regarding prediction accuracy for detailed mechanisms, Sun et al. [19] compared the ignition delay times of the 1-methylnaphthalene detailed chemical kinetic models constructed by Wang et al. and Narayanaswamy et al. under a broader range of conditions, including equivalence ratios from 0.5 to 1.5, pressures between 10 to 40 bar, and temperatures ranging from 1000 to 1400 K. The results indicated that the simulated values from the Wang model were consistently higher than the experimental values at 40 bar, and similarly elevated at 10 bar for equivalence ratios of 1.0 and 1.5. Meanwhile, the Narayanaswamy model’s simulated values were higher than the experimental values across the temperature range of 1000 to 1400 K.

In summary, the current chemical kinetic models of 1-methylnaphthalene exhibit incomplete reaction pathways, hindering a deeper understanding of soot formation in diesel combustion and its subsequent regulation. Additionally, there mechanisms show deviations in the prediction performance, highlighting the need for a more comprehensive and accurate detailed kinetic model.

Based on the above content, this study first analyzes how the rate constants of key reactions in the 1-methylnaphthalene chemical kinetic models affect ignition delay times. A detailed chemical kinetic model for 1-methylnaphthalene is then developed, and its prediction accuracy is validated by comparing the ignition delay times, species concentration profiles of the species, and laminar flame speeds. On this basis, reaction pathway and sensitivity analyses are employed to thoroughly investigate the consumption pathways and patterns of various species during the ignition process of 1-methylnaphthalene.

## 2. Results and Discussion

### 2.1. Validation of Chemical Kinetic Model

This study compared the simulated ignition delay times and the species concentration profiles from the detailed 1-methylnaphthalene mechanism developed in the present study with those from the mechanisms by Wang, Nobili, and Narayanaswamy. Additionally, the present 1-methylnaphthalene mechanism was also validated against the laminar flame speeds and compared with the corresponding modeled results from the study of Nobili et al. [13]. The results indicated that the simulated values from the present 1-methylnaphthalene mechanism closely matched the experimental values.

**Figure 1:**
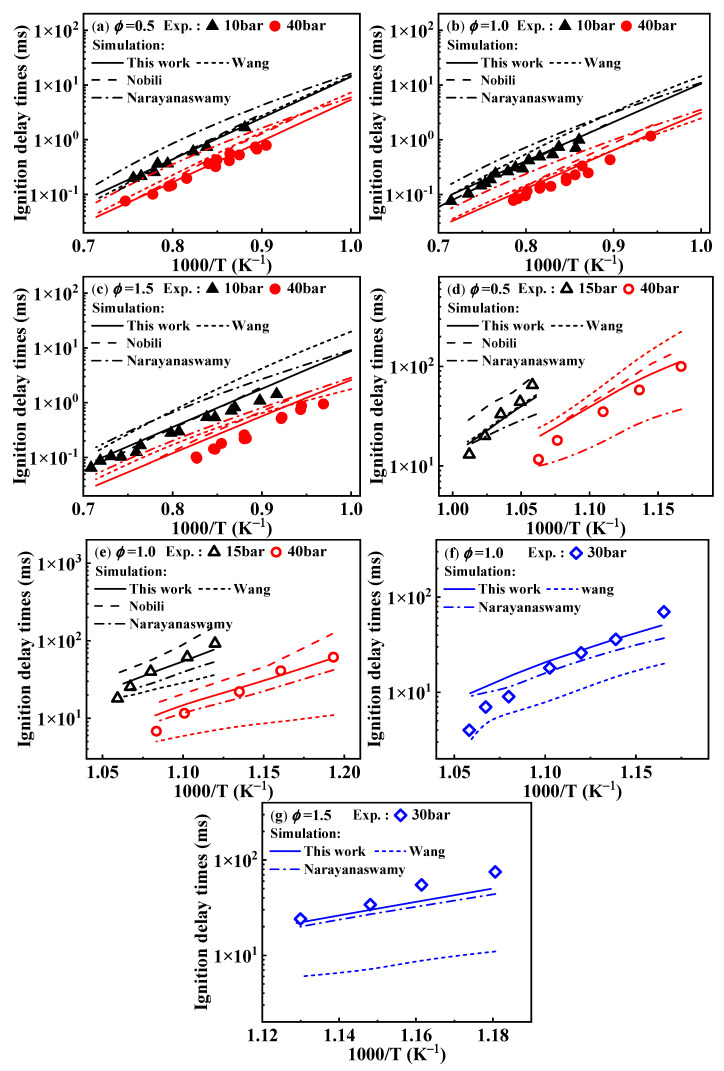
Comparison of experimental ignition delay times of 1-methylnaphthalene and simulated ones from various reaction mechanisms. (**a**–**c**) present ignition delay times corresponding to the ST condition. (**d**–**g**) show ignition delay times corresponding to the RCM condition. Symbols and lines represent experimental data [10,11] and modeled values, respectively. (solid lines: the present mechanism; short dash lines: Wang mechanism [11]; dash lines: Nobili mechanism [13]; dash dot lines: Narayanaswamy mechanism [14]).

Figure 1a–c present the predictions of ignition delay times from the present mechanism under conditions from 1000 to 1400 K. Under these conditions, the simulated values of the mechanism developed in this study are consistent with the experimental values, although the simulated values are slightly higher at *ϕ* = 1.5 and *p* = 40 bar. In the temperature range of 850–950 K, the overall simulated values align with the experimental values; however, deviations arise near 950 K, as shown in Figure 1d–f, where this phenomenon is consistently observed. To explain this issue, the operating principle of the RCM was investigated. The RCM is an experimental device designed to study the combustion chemistry of fuels under high pressure and low to moderate temperature conditions. Since it is challenging to directly measure the compression temperature during the operation of the RCM, researchers use the adiabatic core hypothesis to estimate the compression temperature based on pressure changes. The relationship between the temperature and pressure at the end of compression stroke is described by Equation (1).

(1) $$\int_{T_{0}}^{T_{C}}\frac{\gamma }{\gamma -1}\frac{dT}{T}=\ln\left(\frac{p_{C}}{p_{0}}\right)$$

where *p*_C_ is the measured pressure at the end of compression, *T*_C_ the temperature at the end of compression, *p*_0_ and *T*_0_ the initial pressure and temperature, respectively, and *γ* the temperature-dependent ratio of the mixture.

The higher the compression temperature required for the experiment, the lower the initial pressure must be. Weber et al. [20] noted that the uncertainty of the compression temperature is influenced by the actual value of the initial pressure. In general, lower initial pressure leads to higher compression temperature, which in turn increases the uncertainty in the measured ignition delay times. This observation aligns with the results of their experiments involving methane and cyclohexane [21].

**Figure 2:**
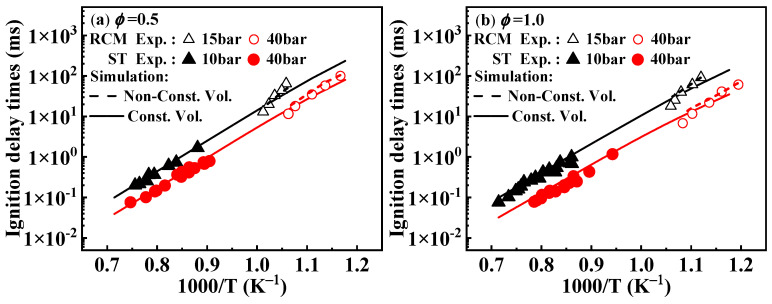
Comparison between experimental ignition delay times of 1-methylnaphthalene from both RCM and ST and the corresponding simulated results from the present mechanism. Symbols and lines indicate experimental data [10,11] and modeled values, respectively.

As shown in Figure 2, when comparing the results under non-constant volume conditions that account for heat loss, the simulated ignition delay times from the present mechanism are higher than those under constant volume conditions, which aligns with theoretical expectations. Around 950 K, the experimental values from the RCM tend to deviate from the overall experimental values, and this deviation from the simulated values can be corroborated by the conclusions drawn by Weber et al. [20].

Regarding the differences in ignition delay times from various mechanisms of 1-methylnaphthalene, the performances of the Wang and Narayanaswamy mechanisms in predicting ignition delay times have been analyzed in the study of Sun et al. [19], as mentioned in the Introduction. Additionally, from Figure 1 it can be seen that the simulated ignition delay times of the Nobili mechanism (sourced from reference [13]) are slightly higher than the experimental data from the shock tube at 40 bar above 1000 K and also higher than the RCM data at 15 and 40 bar below 1000 K. Under these conditions, the modeled results of the Nobili mechanism are generally higher, to some extent, than the ones from the present mechanism.

**Figure 3:**
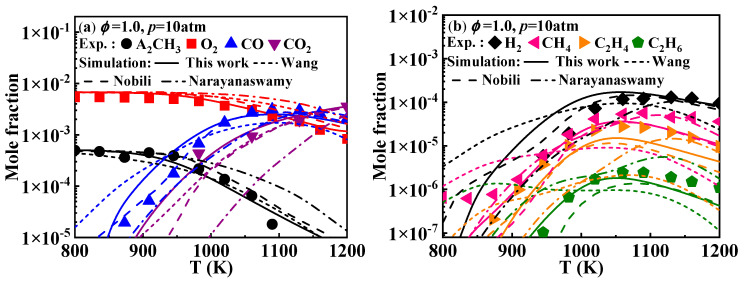
Comparison of experimental key species concentration profiles of 1-methylnaphthalene and simulated ones from various kinetic models. Symbols and lines denote experimental data from the study of Mati et al. [12] and modeled values, respectively. (solid lines: the present mechanism; short dash lines: Wang mechanism [11]; dash lines: Nobili mechanism [13]; dash dot lines: Narayanaswamy mechanism [14]).

Additionally, Mati et al. [12] measured the concentration profiles of the reactants, products, and various stable intermediates during the oxidation of 1-methylnaphthalene in a jet-stirred reactor (JSR). Figure 3 presents the comparison between the simulated and experimental concentrations profiles of key species during the oxidation of 1-methylnaphthalene under the conditions of *ϕ* = 1 and *p* = 10 atm. The detailed mechanisms evaluated include A_2_CH_3_, O_2_, CO, CO_2_, H_2_, CH_4_, C_2_H_4_, and C_2_H_6_. The results indicate that the overall simulated concentrations of the components from the present mechanism align well with the experimental values. Specifically, the predictions for A_2_CH_3_, O_2_, and CO_2_ are accurate. The predictions for CH_4_, C_2_H_4_, and C_2_H_6_ are also reliable, though the simulated values slightly underestimate the experimental values after 1100 K. The predictions for CO and H_2_ are accurate after 1000 K; however, due to increased ignition activity, A_2_CH_3_ is consumed earlier in the oxidation process, leading to a corresponding decrease in the temperature at which CO and H_2_ are produced. A comparison reveals that the species concentration profiles predicted by the Narayanaswamy and Wang mechanisms exhibit deviations from the experimental values. For the Nobili mechanism, the predicted values are generally close to the experimental values. Only for the small molecule hydrocarbon compounds such as CH_4_, C_2_H_4_, and C_2_H_6_ do the modeled results seem to be slightly lower.

**Figure 4:**
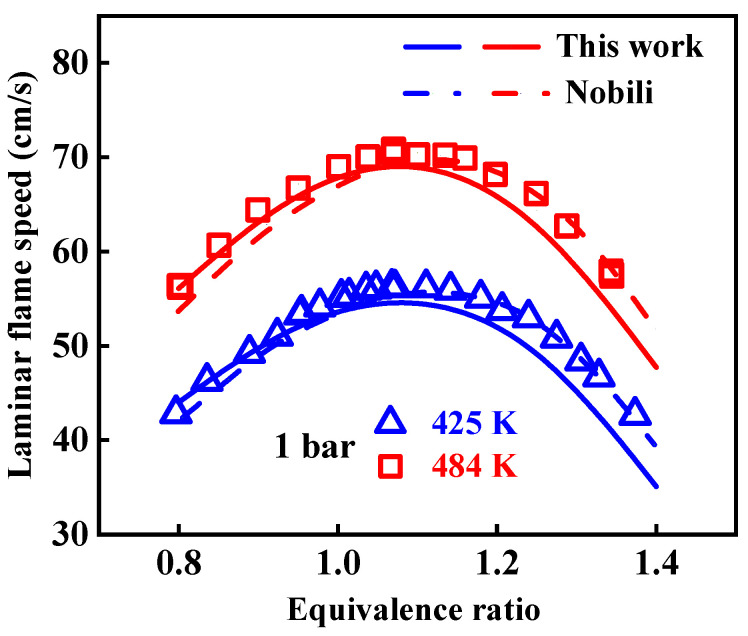
Comparison of experimental laminar flame speeds of 1-methylnaphthalene and simulated results from the present and Nobili kinetic models. Symbols and lines are experimental data in the study of Nobili et al. [13] and modeled values, respectively. (solid lines: the present mechanism; dash lines: modeled values reported in the study of Nobili et al. [13]).

In Figure 4, the present 1-methylnaphthalene mechanism is validated against the experimental laminar flame speeds obtained by Nobili et al. [13] at pressure of 1 bar, initial temperatures from 425 to 484 K, and equivalence ratios between 0.8 and 1.35. The modeled result from the Nobili mechanism is also presented in this figure. The predicted laminar flame speeds of the present mechanism were derived using the PREMIX program [22] in the CHEMKIN-PRO 15131 software. When conducting the simulation, the thermal diffusion was taken into account, and the mixture-averaged method was selected to evaluate the transport properties of species, considering the long calculation time caused by the large scale of the mechanism used. The impact of the grid point on the calculated result of the laminar flame speed was controlled through reducing the values of Gradient and Curvature of the numerical solution. From Figure 4, it is indicated that, on the whole, both of these two mechanisms can predict well the laminar flame speeds of 1-methylnaphthalene, and the Nobili mechanism behaves better than the one developed in the present study. Specifically, the modeled values of the present mechanism are slightly lower than the experimental values at high equivalence ratios, which are also lower than the modeled ones from the Nobili mechanism. While at low equivalence ratios, the modeled values of both mechanisms agree well with the experimental data. The experimental values of laminar flame speeds of 1-methylnaphthalene shown here are the only ones available in the literature, and considering the differences in predicted results of ignition and oxidation between the present and Nobili mechanisms, more experimental data at other conditions may be necessary to validate and improve the 1-methylnaphthalene mechanism.

### 2.2. Chemical Kinetic Analysis of the Ignition of 1-Methylnaphthalene

**Figure 5:**
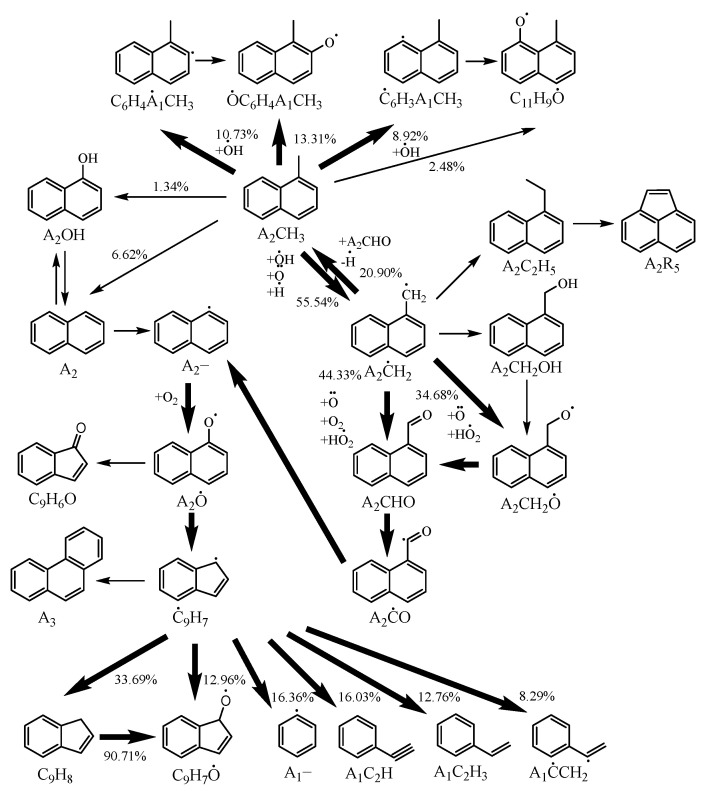
Reaction pathway for 1-methylnaphthalene under conditions of *T* = 1200 K, *ϕ* = 1, *p* = 10 bar with 20% fuel consumption. The reaction paths with more significance are highlighted using lines with greater width.

Figure 5 illustrates the reaction pathway of 1-methylnaphthalene under the conditions of *T* = 1200 K, *ϕ* = 1, *p* = 10 bar with 20% fuel consumption, highlighting the main reaction pathways with thick arrows. It is evident that hydrogen abstraction reactions play a dominant role in the consumption of 1-methylnaphthalene. Among these reactions, the one that generates 1-naphthylmethyl through hydrogen abstraction is the most significant, accounting for 55.54% of the total consumption. This is followed by hydrogen abstraction reactions on the benzene ring of 1-methylnaphthalene, which account for 10.73% and 8.92% of the total consumption, respectively. This pattern is similar to the primary consumption process of 1-methylnaphthalene in the Wang mechanism.

During the consumption process of 1-naphthylmethyl, the formation of 1-naphthaldehyde and 1-naphthylmethyl radical is significant, accounting for 44.33% and 34.68%, respectively. Additionally, 20.9% of the 1-naphthylmethyl reverts to 1-methylnaphthalene, following a reaction pathway similar to that observed in the consumption process of benzyl. Subsequently, 1-naphthaldehyde decomposes into naphthyl radicals, which are then oxidized into naphthoxy radicals before further decomposing into indenyl radicals. The indenyl radicals undergo ring-opening reactions, yielding phenyl, phenylacetylene, and styrenyl, which participate in subsequent reactions.

**Figure 6:**
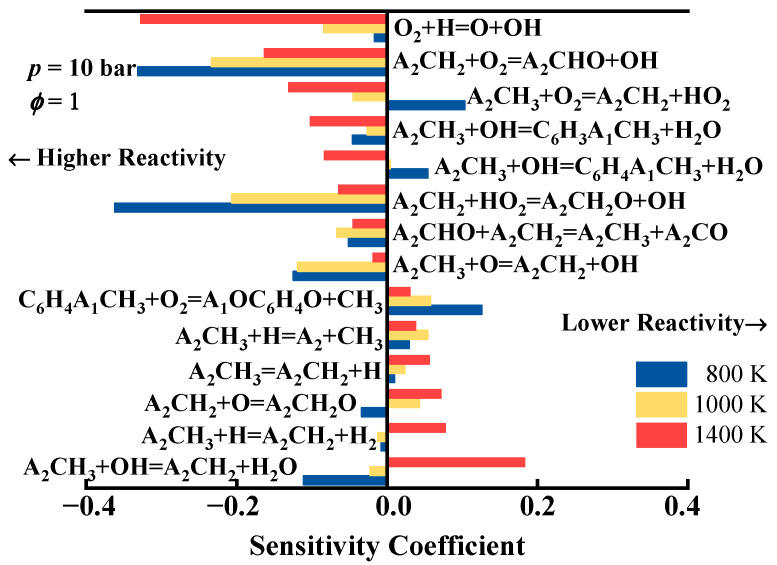
Sensitivity analysis of the ignition delay times using the developed detailed kinetic model of 1-methylnaphthalene.

Figure 6 presents the sensitivity of the ignition delay time using the developed mechanism at *T* = 800, 1000, and 1400 K with *ϕ* = 1 and *p* = 10 bar. It is evident that the hydrogen abstraction reactions of 1-methylnaphthalene and the oxidation reactions of 1-naphthylmethyl significantly influence the ignition delay time. Specifically, the reactions A_2_ĊH_2_ + O_2_ = A_2_CHO + $\;\dot{O}H$ and A_2_ĊH_2_ + ${H\dot{O}}_{2}$ = $A_{2}{\mathrm{CH}}_{2}\dot{O}$ + $\;\dot{O}H$ consistently enhance fuel reactivity at all temperatures, significantly reducing the ignition delay time. The reactions A_2_CH_3_ + $\;\dot{O}H$ = A_2_ĊH_2_ + H_2_O and A_2_CH_3_ + Ḣ = A_2_ĊH_2_ + H_2_ promote ignition at 1000 K; however, as the temperature increases to 1400 K, these reactions start to exhibit a noticeable inhibitory effect on ignition. Conversely, the reaction A_2_CH_3_ + $\;\dot{O}H$ = $C_{6}H_{4}{\dot{A}}_{1}{\mathrm{CH}}_{3}$ + H_2_O promotes fuel ignition at high temperatures but begins to inhibit ignition as the temperature decreases below 1000 K. The reaction A_2_CH_3_ + O_2_ = A_2_ĊH_2_ + ${H\dot{O}}_{2}$ significantly inhibits fuel ignition only at low-temperature conditions (800 K), while at medium to high temperatures (1000–1400 K), it noticeably promotes ignition. Additionally, the chain-propagating reaction of small molecules, O_2_ + Ḣ = Ö + $\dot{O}H$, enhances fuel ignition across all temperatures, exhibiting a particularly pronounced effect at high temperatures.

## 3. Materials and Methods

### 3.1. Chemical Kinetic Model

The detailed mechanisms of large hydrocarbon molecules are typically developed using a hierarchical approach. This includes core mechanisms(C_0_–C_5_) for small molecules, along with fuel-specific sub-mechanisms tailored for various carbon numbers. In each sub-mechanism, reactions can be categorized into multiple reaction classes [23]. The detailed chemical kinetic model of 1-methylnaphthalene in this study comprises a core mechanism and a fuel sub-mechanism for 1-methylnaphthalene. For the core mechanism, NUIG-mech1.3 [24] was selected, as it has recently been updated with improved reaction pathways for hydrogen and methane. Additionally, the rate constant for the chain termination reaction Ḣ + O_2_ (+M) = ${H\dot{O}}_{2}$(+M) has been revised based on experimental data.

The fuel sub-mechanism was developed by reviewing existing chemical kinetic models for 1-methylnaphthalene, similar aromatic hydrocarbon fuels, and their derivatives. Considering the ignition process, the fuel sub-mechanism constructed in this study encompasses hydrogen abstraction reactions, substitution reactions, and high-temperature decomposition of the fuel. It also includes oxidation reactions of 1-naphthylmethyl, reverse reactions to 1-methylnaphthalene, addition reactions with radicals, and isomerization reactions. Additionally, the model addresses the oxidation and decomposition of 1-naphthylmethyl-oxy ($A_{2}{\mathrm{CH}}_{2}\dot{O}$) and naphthaldehyde (A_2_CHO), as well as conversions among naphthalene, naphthoxy, and naphthol, including their decomposition. Lastly, it considers reactions between indene and indenyl species, along with conversions to monocyclic compounds. The specific reaction classes and key reactions are shown in Table 1. Then, this study discusses the rate constant of each reaction class and adjusts the rate constants of certain reactions. The main adjusted reactions and their recommended rate constants are shown in Table 2. Reaction rate constant adjustments are implemented by modifying the pre-exponential factor *A*. In this study, all rate constant adjustments range between 0.5 and 2 times the reaction rate constants derived from calculations, experiments, or analogies in the reference. The process for determining the data in Table 2 can be found in detail in Section 3.2, Section 3.3, Section 3.4 and Section 3.5 below. The detailed 1-methylnaphthalene mechanism developed in this study contains 1389 species and 7185 reactions. The thermodynamic data were primarily obtained from the Burcat database [25], while the thermodynamic data for certain aromatic hydrocarbon derivatives were derived from calculations by Bounaceur [17] and Blanquart [18]. The mechanism as well as the thermodynamic and transport data can be found in the Supplementary Material.

In addition, the modeled values of ignition delay times and species concentration profiles involved were derived using the CHMEKIN-PRO 15131 software. Specifically, the ignition delay times are modeled with the zero-dimensional, homogeneous, adiabatic assumption. The perfectly stirred reactor (PSR) in the CHMEKIN-PRO 15131 software was utilized to predict the concentration profiles of concerned species.

### 3.2. Rate Constants of Reactions of 1-Methylnaphthalene

#### 3.2.1. Hydrogen Abstraction of 1-Methylnaphthalene

**Figure 7:**
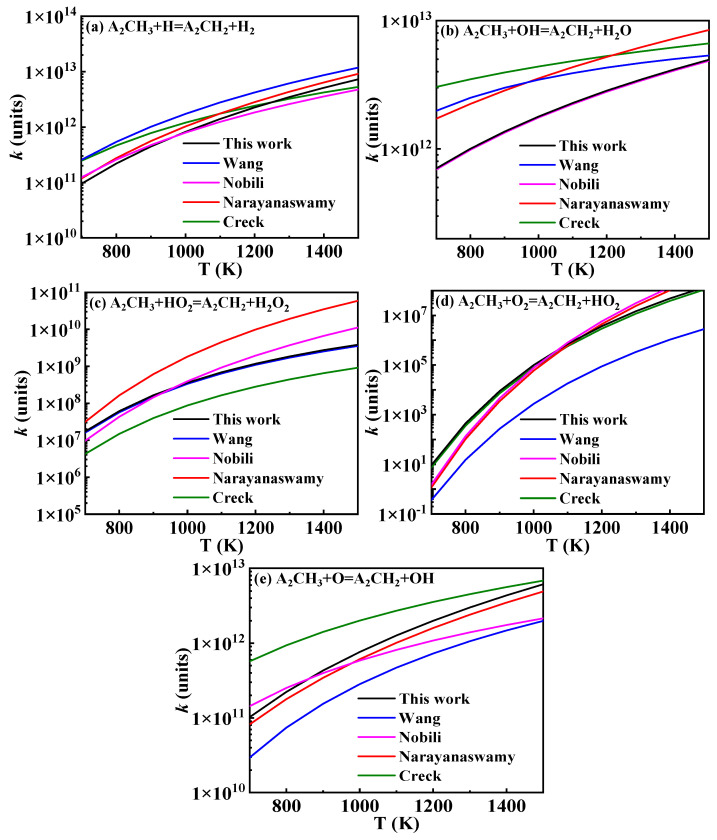
Comparison of rate constants for hydrogen abstraction reactions of 1-methylnaphthalene from different sources (black lines: the present mechanism; blue lines: Wang mechanism [11]; pink lines: Nobili mechanism [13]; red lines: Narayanaswamy mechanism [14]; green lines: Creck mechanism [15]).

The hydrogen abstraction reactions of A_2_CH_3_ serve as the primary pathways for fuel consumption and play a crucial role in both fuel ignition and oxidation. The existing hydrogen abstraction reaction rate constants for the methyl group on A_2_CH_3_ are illustrated in Figure 7. In the Wang mechanism, the reaction rate constants are sourced from Mati’s work, which is based on the hydrogen abstraction rate constants of toluene. These rate constants were obtained by Emdee et al. [26] through the QRRK calculation method [27]. The reaction rate constants in the Narayanaswamy mechanism were similarly derived by analogy to the hydrogen abstraction of toluene, with rate constants calculated by Seta [28] using transition state theory (TST) and the B3LYP/6-31G(d) method. Additionally, the rate constants analyzed in the Creck and Nobili mechanisms are included. The rate constants selected for this study are indicated by the black lines in Figure 7.

By comparing the hydrogen abstraction reactions of A_2_CH_3_ in the various 1-methylnaphthalene mechanisms, it was found that in the Mati and Narayanaswamy mechanisms, the radicals initiating the reactions are primarily small molecular radicals. In contrast, the Wang and Creck mechanisms consider a greater variety of large molecular radicals in the hydrogen abstraction reactions of A_2_CH_3_, derived by analogy to the hydrogen abstraction reactions of toluene. The Creck mechanism also involves additional small molecular radicals. In this study, by drawing an analogy to the corresponding reactions of toluene, small molecular radicals involved in the hydrogen abstraction reactions of 1-methylnaphthalene are considered up to C_4_. Meanwhile, the large molecular radicals include important intermediate radicals generated during the reaction processes of 1-methylnaphthalene, such as ${\;\dot{O}C}_{6}H_{4}A_{1}{\mathrm{CH}}_{3}$ (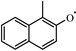), $A_{2}{\mathrm{CH}}_{2}\dot{O}$, A_2_−, Ċ_9_H_7_, A_1_CHĊH, $A_{1}{\mathrm{CH}}_{2}\dot{O}$, and A_1_−.

In addition to hydrogen abstraction from the methyl group, Wang et al. studied the hydrogen abstraction reactions occurring on the aromatic ring of A_2_CH_3_. They confirmed the significance of this reaction class through reaction pathway analysis, demonstrating that it accounts for 18% of all consumption pathways for A_2_CH_3_. Similarly, this study incorporates these reactions, with reaction rate constants derived from Wang’s mechanism, which analogized Bounaceur’s work on the hydrogen abstraction reaction rate constants of the toluene ring. Furthermore, this study includes hydrogen abstraction reactions on the aromatic ring of A_2_CH_3_ with O_2_ that are absent in the Wang mechanism, such as A_2_CH_3_ + O_2_ = $C_{6}H_{4}{\dot{A}}_{1}{\mathrm{CH}}_{3}$ + ${H\dot{O}}_{2}$ and A_2_CH_3_ + O_2_ = Ċ_6_H_3_A_1_CH_3_ + ${H\dot{O}}_{2}$. The rate constant of the hydrogen abstraction reaction A_2_CH_3_ + $\;\dot{O}H$ = $C_{6}H_{4}{\dot{A}}_{1}{\mathrm{CH}}_{3}$ + H_2_O has also been adjusted.

**Figure 8:**
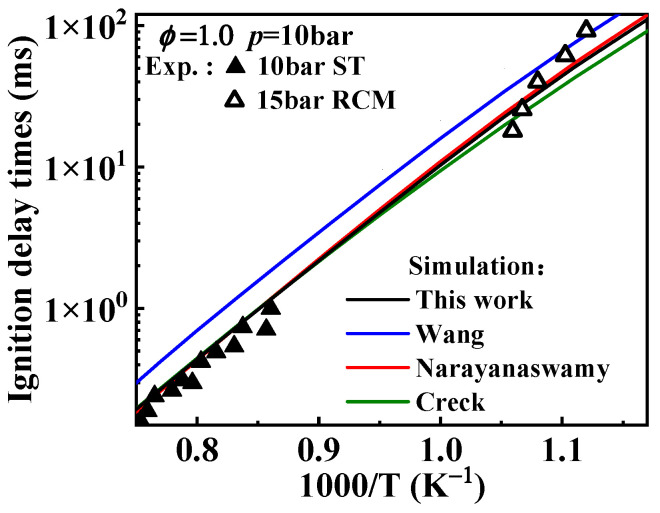
Comparison between experimental ignition delay times of 1-methylnaphthalene and simulated ones from the mechanisms with different rate constants of the hydrogen abstraction reactions of 1-methylnaphthalene. Symbols and lines indicate experimental data [10,11] and modeled values, respectively. (black line: the present mechanism; blue line: Wang mechanism [11]; red line: Narayanaswamy mechanism [14]; green line: Creck mechanism [15]).

The predicted ignition delay times for 1-methylnaphthalene under conditions of *ϕ* = 1 and *p* = 10 bar are displayed in Figure 8. In the hydrogen abstraction reactions of A_2_CH_3_, the reaction between A_2_CH_3_ and $\;\dot{O}H$ serves as the primary consumption pathway. According to the trend of reaction rate constants in Figure 7b, the rate constants in the Creck mechanism are higher than those in the Wang mechanism. In addition, the rate constants in the Narayanaswamy mechanism surpass those of the Creck mechanism at temperatures above 1200 K, while remaining slightly lower than Wang rate constants below 950 K. Consequently, the influence of A_2_CH_3_ hydrogen abstraction rate constants on ignition delay times is expected to show a similar trend in speed. However, in this reaction class, apart from the hydrogen abstraction reactions with Ḣ atoms, the rate constants for the reactions in the Wang mechanism are lower than those in the Narayanaswamy mechanism, leading to slower ignition at lower temperatures under the Wang rate constants. Figure 8 shows the simulated results for ignition delay times under different 1-methylnaphthalene hydrogen abstraction rate constants, and it can be seen that the ignition delay times predicted with Wang rate constants are longer under 800–1400 K. The adjusted mechanism shows that the hydrogen abstraction reaction rate constants of 1-methylnaphthalene with $\;\dot{O}H$, Ö and Ḣ radicals are similar to those of Narayanaswamy mechanism, and these radicals are more reactive. Therefore, the predicted ignition delay times from the mechanism with adjusted hydrogen abstraction reaction rate constants of 1-methylnaphthalene in this study are closer to those predicted based on the Narayanaswamy rate constants.

#### 3.2.2. Other Reaction Classes of 1-Methylnaphthalene

In addition to the hydrogen abstraction reactions of 1-methylnaphthalene, its consumption pathways also include substitution reactions and decomposition reactions. Substitution reactions significantly influence ignition delay times at high pressures, whereas decomposition reactions play a crucial role in high-temperature ignition.

**Figure 9:**
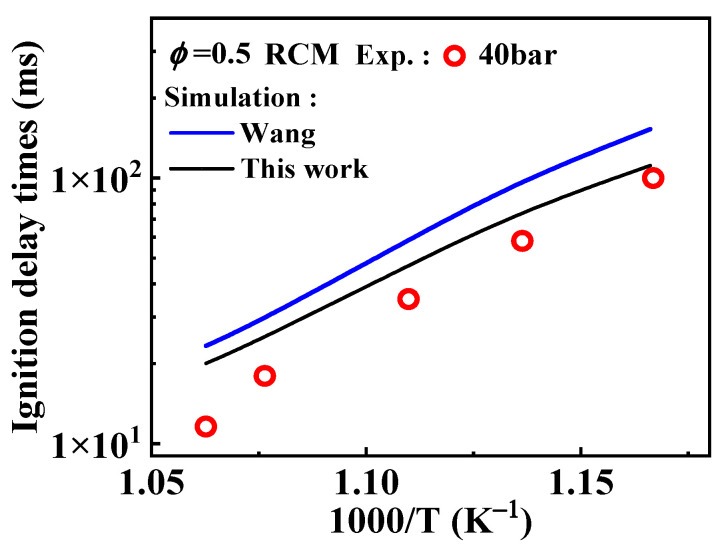
Comparison of experimental ignition delay times and the corresponding predicted values from the kinetic models with different selected rate constants for relevant reactions during the oxidation of 1-methylnaphthalene. Symbols and lines are experimental data [10] and modeled values, respectively. (black line: the present mechanism; blue line: Wang mechanism [11]).

Among the existing mechanisms, only the Wang mechanism has studied the substitution reactions of 1-methylnaphthalene, using reaction rate constants obtained by analogy to those of toluene. These rate constants for toluene were determined through experimental studies conducted by Bounaceur et al. [16]. The substitution reactions of A_2_CH_3_ with Ö and $\;\dot{O}H$ radicals occur at the ring positions, resulting in the formation of ${\;\dot{O}C}_{6}H_{4}A_{1}{\mathrm{CH}}_{3}$ and $C_{11}H_{9}\;\dot{O}$ (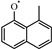). Additionally, the substitution of a Ḣ atom with an $\;\dot{O}H$ radical at the same positions produces HOC_6_H_4_A_1_CH_3_ and C_11_H_9_OH. The formation and consumption of ${\;\dot{O}C}_{6}H_{4}A_{1}{\mathrm{CH}}_{3}$ and $C_{11}H_{9}\;\dot{O}$ significantly influence the simulations under conditions of *ϕ* = 0.5 and *p* = 40 bar with low temperatures. In this study, the rate constants of the following reactions from the Wang mechanism were adjusted: A_2_CH_3_ + Ö = $C_{11}H_{9}\;\dot{O}$ + Ḣ, A_2_CH_3_ + Ö = ${\;\dot{O}\;C}_{6}H_{4}A_{1}{\mathrm{CH}}_{3}$ + Ḣ, and $C_{11}H_{9}\;\dot{O}$ = Ċ_10_H_9_ (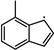) + CO, as shown in Table 2. From Figure 9, it can be observed that after the adjustments, the simulated ignition delay times for 1-methylnaphthalene under *ϕ* = 0.5 and *p* = 40 bar significantly decrease, bringing them closer to the experimental values. Additionally, the methyl group of A_2_CH_3_ can undergo substitution reactions with $\;\dot{O}\;H$ and Ḣ to form A_2_OH and A_2_, respectively. However, these two reaction classes contribute only a small fraction to the overall consumption of 1-methylnaphthalene.

### 3.3. Rate Constants of Reactions of 1-Naphthylmethyl

#### 3.3.1. Oxidation of 1-Naphthylmethyl

**Figure 10:**
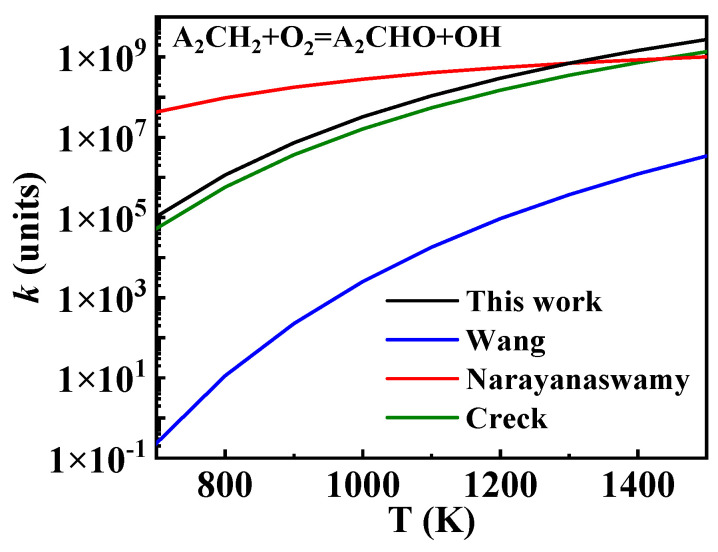
Comparison of rate constants for the reaction A_2_ĊH_2_ + O_2_ = A_2_CHO + $\;\dot{O}H$ from different sources. (black line: the present mechanism; blue line: Wang mechanism [11]; red line: Narayanaswamy mechanism [14]; green line: Creck mechanism [15]).

The oxidation of A_2_ĊH_2_ is a crucial pathway in the consumption of A_2_CH_3_, as most A_2_ĊH_2_ progresses through this reaction to form other intermediate products of 1-methylnaphthalene. This is one of the key reaction classes that significantly influences the performance of the kinetic model, playing a crucial role in the ignition and oxidation of 1-methylnaphthalene. A key difference in the oxidation of A_2_ĊH_2_ among existing 1-methylnaphthalene mechanisms is found in the formation and consumption of $A_{2}{\mathrm{CH}}_{2}\;\dot{O}$. In the Creck mechanism, the formation and consumption of this species are absent, with A_2_ĊH_2_ primarily converting to A_2_CHO. Similarly, in the Wang mechanism, A_2_ĊH_2_ mainly forms A_2_CHO. Although $A_{2}{\mathrm{CH}}_{2}\;\dot{O}$ is included, it is generated through the hydrogen abstraction reaction of A_2_CH_2_OH with O_2_, and both this reaction and A_2_CH_2_OH itself contribute very little. The reaction rate constants for this part were also derived from Bounaceur et al. [16]. However, in the Narayanaswamy mechanism, $A_{2}{\mathrm{CH}}_{2}\;O˙$ is equally important asA_2_CHO as an oxidation product of A_2_ĊH_2_, and $A_{2}{\mathrm{CH}}_{2}\;O\;˙$ subsequently reacts to form A_2_CHO. To understand this difference, a comparison was made to the oxidation of benzyl (A_1_ĊH_2_), revealing that the Narayanaswamy mechanism reflects a similar relationship between the oxidation of A_1_ĊH_2_ to $A_{1}{\mathrm{CH}}_{2}\;\dot{O}$ and A_1_CHO. Therefore, in this study, the oxidation of A_2_ĊH_2_ to $A_{2}{\mathrm{CH}}_{2}\;\dot{O}$ was considered, with rate constants taken from the Wang and Narayanaswamy mechanisms for comparison. Additionally, the shared reaction rate constant of A_2_ĊH_2_ + O_2_ = A_2_CHO + $\;\dot{O}H$, shown in Figure 10, was selected from the Creck mechanism. The rate constants of several key reactions, including this one, were adjusted, as detailed in Table 2.

**Figure 11:**
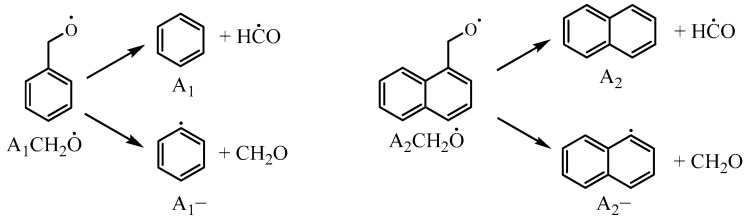
Decomposition reactions of benzyloxy and 1-naphthylmethyloxy radicals.

In the decomposition of $A_{2}{\mathrm{CH}}_{2}\dot{O}$, two different reaction pathways exist: $A_{2}{\mathrm{CH}}_{2}\dot{O}$ = A_2_− + CH_2_O and $A_{2}{\mathrm{CH}}_{2}\dot{O}$ = A_2_ + HĊO. In the Narayanaswamy mechanism, the decomposition predominantly favors the former pathway. The decomposition of $A_{1}{\mathrm{CH}}_{2}\dot{O}$ produce both benzene + formyl (HĊO) and phenyl + formaldehyde (CH_2_O). Therefore, this study incorporated the reaction pathway for $A_{2}{\mathrm{CH}}_{2}\dot{O}$ to produce naphthalene and formyl, as shown in Figure 11, and compared it to the reaction rate constants for $A_{1}{\mathrm{CH}}_{2}\dot{O}$ to produce benzene and formyl in the Narayanaswamy mechanism. Additionally, the reaction rate constant for $A_{2}{\mathrm{CH}}_{2}\dot{O}$ = A_2_CHO + Ḣ was also adjusted in this study.

**Figure 12:**
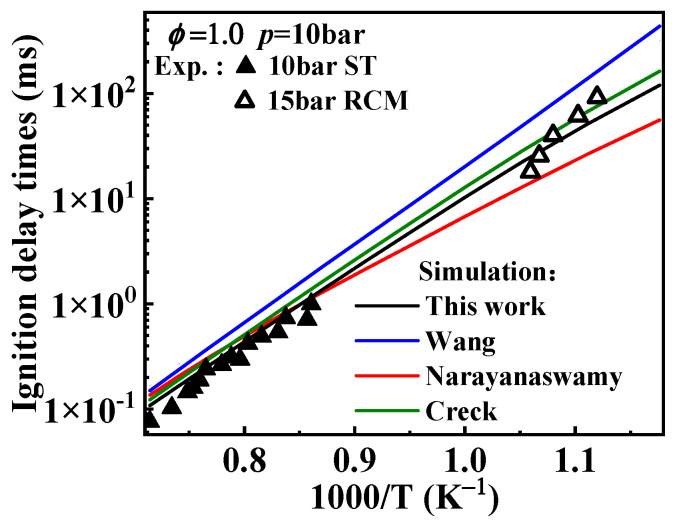
Comparison between the experimental ignition delay times and the simulated values derived from the mechanisms with different selected rate constants for the oxidation reactions of 1-naphthylmethyl. Symbols and lines denote experimental data [10,11] and modeled values, respectively. (black line: the present mechanism; blue line: Wang mechanism [11]; red line: Narayanaswamy mechanism [14]; green line: Creck mechanism [15]).

The selection of rate constants for the oxidation of A_2_ĊH_2_ and the consumption of its oxidation product, $A_{2}{\mathrm{CH}}_{2}\dot{O}$, significantly enhanced the fuel’s reactivity. Figure 12 compares the predicted ignition delay times, after adjusting the rate constants for these key reactions, with those from different mechanisms. It can be observed that the ignition delay times predicted by this study’s mechanism are significantly reduced across the entire temperature range. The Wang rate constants exhibit similar behavior in predicting ignition delay times based on the rate constants of A_2_CH_3_ hydrogen abstraction reaction, where lower reaction rate constants result in higher ignition delay times. The Narayanaswamy rate constants predict much shorter ignition delay times at lower temperatures, while above 1250 K, the predictions do not show significant difference compared to those of the Creck rate constants.

#### 3.3.2. Other Reaction Classes of 1-Naphthylmethyl

The conversion of 1-naphthylmethyl to 1-methylnaphthalene represents a significant proportion of its consumption, particularly under high-temperature conditions. The Wang mechanism proposes reaction rate constants for this process, which are derived from the same sources as the hydrogen abstraction reactions of 1-methylnaphthalene. Although the isomerization of 1-naphthylmethyl and its addition reactions with radicals account for a relatively small portion of the overall combustion process of 1-methylnaphthalene, they are important pathways for the formation of PAHs during combustion. This process slows down the combustion, and both Jin et al. and Mati et al. have studied the rate constants associated with this class of reactions. The isomerization rate constants were derived by analogy to those calculated by Jasper et al. [29] using Ab initio, TST, and ME methods for the isomerization between fulvene and benzene. The rate constants for the addition reactions with radicals were based on the benzyl-related reaction rate constants calculated by Brand et al. [30] using the Statistical Adiabatic Channel Model (SACM).

**Figure 13:**
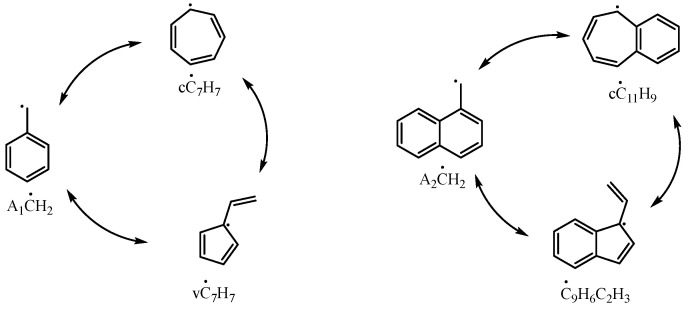
Isomerization reactions of benzyl and 1-naphthylmethyl radicals.

A_2_ĊH_2_ can be reconverted to A_2_CH_3_ by reacting with Ḣ atoms from other intermediate products of 1-methylnaphthalene, as seen in the reactions A_2_ĊH_2_ + A_2_CHO = A_2_CH_3_ + A_2_ĊO and A_2_ĊH_2_ + A_2_OH = A_2_CH_3_ + $A_{2}\dot{O}$. Under high-temperature conditions (above 1000 K), A_2_ĊH_2_ can also directly react with a Ḣ atom to form A_2_CH_3_ through the reaction A_2_ĊH_2_ + Ḣ = A_2_CH_3_. As the temperature increases, this reaction increasingly contributes to the consumption of A_2_ĊH_2_. Additionally, A_2_ĊH_2_ can be consumed through other pathways, including reactions with radicals to form new compounds, isomerizing into different substances, or direct decomposition into low-carbon molecules. Although these reactions contribute only a small portion to the overall flux in the 1-methylnaphthalene reaction process, the combination of A_2_ĊH_2_ with radicals represents an important pathway for converting 1-methylnaphthalene into other polycyclic aromatic hydrocarbons. Among these, the reactions A_2_ĊH_2_ + ĊH_3_ = A_2_C_2_H_5_ and A_2_ĊH_2_ + $\;\dot{O}H$ = A_2_CH_2_OH were proposed by Mati et al. [10]. A_2_C_2_H_5_ influences the formation and consumption of ethyl-naphthalene, vinyl-naphthalene, A_2_ĊHCH_3_, and A_2_CH_2_ĊH_2_ radicals, while A_2_CH_2_OH further reacts to produce $A_{2}{\mathrm{CH}}_{2}\dot{O}$ and A_2_CHO, both of which are key intermediates in the main consumption pathways of A_2_ĊH_2_. Additionally, the isomerization of the 1-naphthylmethyl radical has been investigated by Jin et al. [16]. By drawing parallels to the experimentally verified isomerization between benzyl and C_7_ ring-like species (cĊ_7_H_7_, vĊ_7_H_7_), they proposed a similar isomerization process of 1-naphthylmethyl, as illustrated in Figure 13. The detailed kinetic model in this study incorporates these reactions.

**Figure 14:**
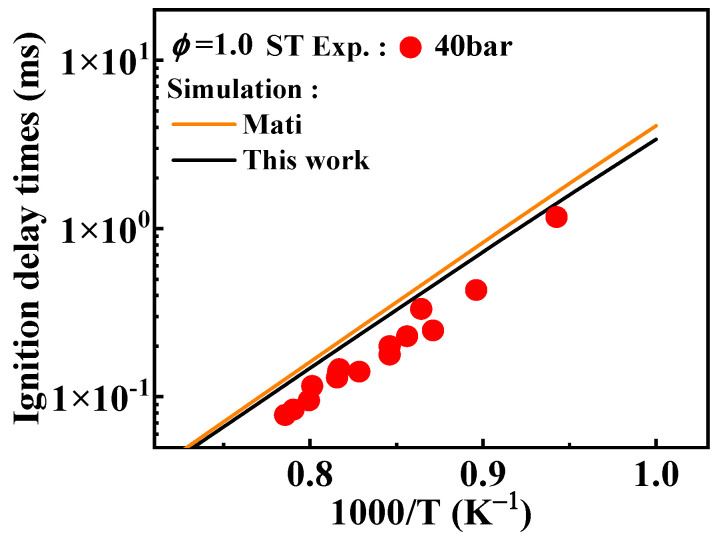
Comparison of simulated ignition delay times from the mechanisms with different selected rate constants for the radical recombination reactions of A_2_ĊH_2_ and the corresponding experimental values [11]. (black line: the present mechanism; orange line: Mati mechanism [12]).

This study adjusted the rate constants for the following reactions: A_2_ĊH_2_ + ĊH_3_ = A_2_C_2_H_5_, A_2_ĊH_2_ + $\;\dot{O}H$ = A_2_CH_2_OH, A_2_CH_2_OH + O_2_ = $A_{2}{\mathrm{CH}}_{2}\dot{O}$ + ${H\dot{O}}_{2}$, and A_2_CH_2_OH + O_2_ => A_2_CHO + ${H\dot{O}}_{2}$ + Ḣ, as detailed in Table 2. Figure 14 shows that these adjustments to the reaction rate constants resulted in a reduction of the ignition delay times of 1-methylnaphthalene for 40 bar.

### 3.4. Rate Constants of Reactions of Indene and Indenyl

In the main consumption pathways of 1-methylnaphthalene, indene (C_9_H_8_) and indenyl (Ċ_9_H_7_) serve as representative species in the process of converting bicyclic structures into monocyclic structures. This class of reactions is crucial for the formation of small molecules during combustion. Currently, reaction rate constants for this process have been calculated in the Wang, Narayanaswamy, Creck, and NUIG-mech1.3 mechanisms.

**Figure 15:**
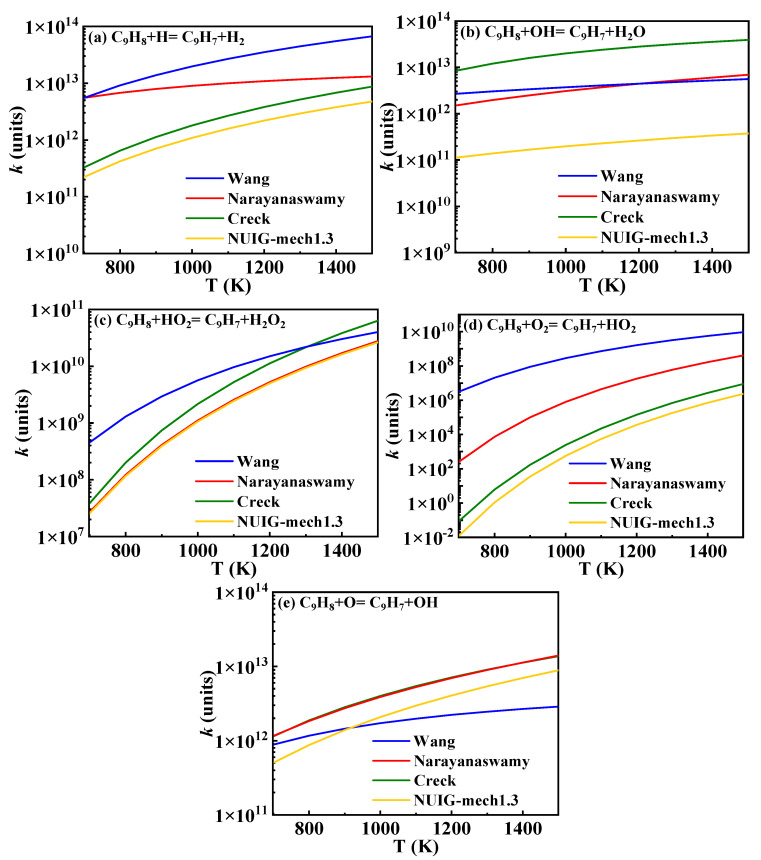
Comparison of rate constants for hydrogen abstraction reactions of indene from different sources. (blue lines: Wang mechanism [11]; red lines: Narayanaswamy mechanism [14]; green lines: Creck mechanism [15]; yellow lines: NUIG-mech1.3 [24]).

The bicyclic structure of 1-methylnaphthalene primarily breaks down into indenyl, indene-oxy ($C_{9}H_{7}\dot{O}$), and indanone (C_9_H_6_O), resulting in the formation of benzene derivatives such as styrenyl, phenyl, and phenylacetylene. These compounds subsequently undergo reactions to form small molecules, highlighting the importance of this class of reactions. The reaction rate constants for the hydrogen abstraction of indene to form indenyl are shown in Figure 15. This study utilizes the rate constants from the Narayanaswamy mechanism. The reactions of indene and indenyl are modeled by analogy to the C_5_ chemical kinetics model. The mechanism presented in this study incorporates the formation and consumption of indene-oxy, as proposed in the Wang mechanism, as well as the formation and consumption of indanone from the Narayanaswamy mechanism, both of which have been validated in the study by Jin et al. [16].

**Figure 16:**
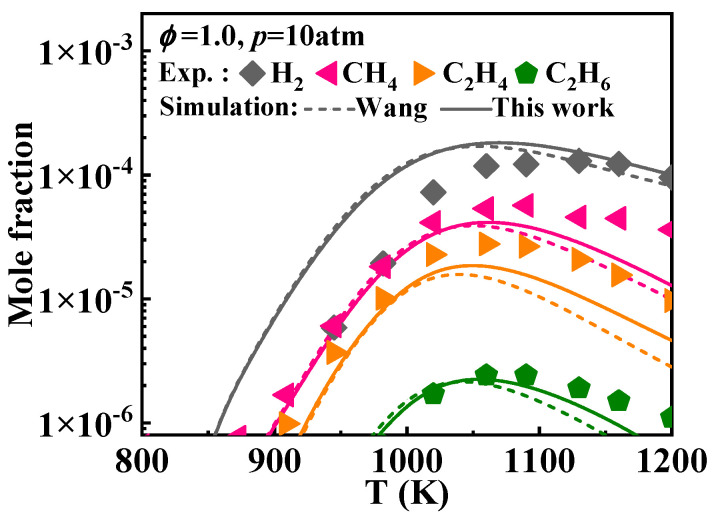
Comparison of simulated concentration profiles of small molecular species as a function of temperature from the mechanisms with various selected rate constants of certain reactions between indene and indenyl and the corresponding experimental values [12]. (solid lines: the present mechanism; short dash lines: Wang mechanism [11]).

This study adjusted some of the reaction rate constants for the interconversion of indene, indenyl, and indene-oxy. This includes the reactions between indene and indenyl (Ċ_9_H_7_ + Ḣ = C_9_H_8_) and the reactions leading to the formation of indene-oxy (Ċ_9_H_7_ + ${H\dot{O}}_{2}$ = $C_{9}H_{7}\dot{O}$ + $\;\dot{O}H$), as detailed in Table 2. This, along with the oxygenation of naphthalene, elevated the concentrations of small molecules above 1050 K during the oxidation of 1-methylnaphthalene, as illustrated in Figure 16.

### 3.5. Rate Constants of Reactions of Naphthalene

In addition to the reaction classes discussed above, the formation and consumption of naphthalene (A_2_) also occur during the combustion of 1-methylnaphthalene, though the flux of these reactions is generally minimal. This study focused solely on adjusting the reaction rate constant for the oxygen addition to naphthalene (A_2_ + Ö = A_2_OH). This adjustment, along with the modifications to the reaction rate constants for indene and indenyl discussed in Section 3.4, collectively enhances the concentration profiles of small molecular species during the oxidation of 1-methylnaphthalene at temperatures above 1050 K.

## 4. Conclusions

In this study, reaction pathways and reaction classes of 1-methylnaphthalene were investigated, and the effects of reaction rate constants from existing detailed mechanisms on ignition delay times were compared. Based on the above research, a detailed kinetic model of 1-methylnaphthalene was constructed. Then, reaction paths and sensitivity analysis were conducted for the ignition of 1-methylnaphthalene. The main conclusions are as follows:

(1) Reactions on the ring of 1-methylnaphthalene, isomerization of 1-naphthylmethyl, formation and consumption of polycyclic aromatic hydrocarbons such as acenaphthene, phenanthrene, and pyrene, as well as formation and consumption of 1-naphthylmethyl-oxy, indenyl-oxy, and indanone were all taken into account in the reaction pathway of 1-methylnaphthalene.
(2) The rate constants of reactions involving 1-methylnaphthalene and its intermediate species were discussed, including hydrogen abstraction, decomposition, and substitution reactions of 1-methylnaphthalene, followed by post-substitution decomposition. Additionally, the oxidation, decomposition, and addition reactions of 1-naphthylmethyl were examined, along with the oxidation of the resulting products. Further reactions discussed include the oxidation of naphthalene, hydrogen abstraction, oxidation, and decomposition of indene, and the oxidation of the indenyl radical. Specific recommended reaction rate constant values were provided for these reactions.
(3) The newly developed chemical kinetic model of 1-methylnaphthalene was comprised of 1389 species and 7185 reactions. This kinetic model was validated against the ignition delay times of 1-methylnaphthalene under equivalence ratios of 0.5, 1.0, and 1.5, pressures ranging from 10 to 40 bar, and temperatures between 800 and 1400 K and the concentration profiles at an equivalence ratio of 1.0, pressure of 10 atm, and temperatures between 800 and 1200 K. Additionally, the present mechanism was also validated against the laminar flame speeds at pressure of 1 bar, initial temperature from 425 to 484 K, and equivalence ratio between 0.8 and 1.35, and the result shows a good agreement was achieved.
(4) The reaction pathway and sensitivity analyses of the ignition process of 1-methylnaphthalene at an equivalence ratio of 1 and a pressure of 10 bar revealed that the conversion of 1-methylnaphthalene to 1-naphthaldehyde through processes such as hydrogen abstraction and oxidation plays an important role in the ignition of 1-methylnaphthalene.

## Figures and Tables

**Table 1:** Reaction classes and key reactions discussed in this study for 1-methylnaphthalene.

Species	Reaction Class	Key Reactions
1-Methylnaphthalene	Hydrogen abstraction	$A_{2}{\mathrm{CH}}_{3}\;+\dot{R}\to A_{2}\dot{C}H_{2}+\mathrm{RH};A_{2}{\mathrm{CH}}_{3}\;+\dot{R}\to$A_2_−CH_3_ + RH
	Substitution	$A_{2}{\mathrm{CH}}_{3}+Ö/\dot{O}H$$\to {\;\dot{O}A}_{2}{\mathrm{CH}}_{3}$A_2_CH_3_/HOA_2_CH3 + Ḣ
	Decomposition	A_2_CH_3_→A_2_ĊH_2_ + Ḣ; A_2_CH_3_→A_2_− + ĊH_3_
1-Naphthylmethyl	Oxidation	$A_{2}ĊH_{2}+O_{2}\to A_{2}{\mathrm{CH}}_{2}\dot{O}$ $/A_{2}\mathrm{CHO}+Ö/\dot{O}H$
	reverse reactions	$A_{2}\dot{C}H_{2}+\mathrm{RH}\to A_{2}{\mathrm{CH}}_{3}\;+\dot{R}$
	Addition	$A_{2}\dot{C}H_{2}+\dot{R}$→A_2_CH_2_R
	Isomerization	A_2_ĊH_2_ = cĊ_11_H_9_ = Ċ_9_H_6_C_2_H_3_
Indenyl	conversion of indene to indenyl	C_9_H_8_ + $\dot{R}$→Ċ_9_H_7_ + RH
Naphthalene	Oxidation	A_2_ + Ö = A_2_OH

**Table 2:** Key reactions and recommended rate constants in the 1-methylnaphthalene mechanism. Rate constant in Arrhenius form (*k* = *AT^n^*exp(-*E*/*RT*)); units are cm^3^, K, mol, s, and kJ, *A*_0_ is from reference.

Reaction Classes	Key Reactions	*A*	*A*_0_ *×*	*n*	*E*	Source
Hydrogen abstraction of 1-methylnaphthalene	$A_{2}{\mathrm{CH}}_{3}+O_{2}=A_{2}ĊH_{2}+{H\dot{O}}_{2}$	2.40 × 10^14^	1.2	0	43,000	Creck [15]
	$A_{2}{\mathrm{CH}}_{3}+Ö=A_{2}ĊH_{2}+\;\dot{O}H$	1.48	1.2	4.09	2545	Narayanaswamy [14]
	$A_{2}{\mathrm{CH}}_{3}+\;\dot{O}H$ = C_6_H_4_A_1_CH_3_ + H_2_O	9.60 × 10^7^	1.2	1.42	1450	Wang [11]
	$A_{2}{\mathrm{CH}}_{3}+{\;\dot{O}C}_{6}H_{4}A_{1}{\mathrm{CH}}_{3}$ = A_2_ĊH_2_ + HOC_6_H_4_A_1_CH_3_	1.92 × 10^11^	1.2	0	15,100	Wang [11]
Decomposition of 1-methylnaphthalene	A_2_CH_3_ = A_2_ĊH_2_ + Ḣ	6.25 × 10^17^	0.5	−0.6	94,787	Narayanaswamy [14]
Substitution reactions of 1-methylnaphthalene and post-substitution decomposition	$A_{2}{\mathrm{CH}}_{3}+Ö={\;\dot{O}C}_{6}H_{4}A_{1}{\mathrm{CH}}_{3}$ + Ḣ	1.73 × 10^13^	2.0	0	3600	Wang [11]
Substitution reactions of 1-methylnaphthalene and post-substitution decomposition Oxidation of 1-naphthylmethyl	$A_{2}{\mathrm{CH}}_{3}+Ö=C_{11}H_{9}\dot{O}$ + Ḣ	4.33 × 10^12^	0.5	0	3600	Wang [11]
	$C_{11}H_{9}\dot{O}$ = Ċ_10_H_9_ + CO	6.00 × 10^11^	2.0	0	43,800	Wang [11]
	$A_{2}ĊH_{2}+{H\dot{O}}_{2}$ $=A_{2}{\mathrm{CH}}_{2}\dot{O}$ $+\;\dot{O}H$	2.38 × 10^9^	2.0	1.03	−2249	Narayanaswamy [14]
Oxidation of 1-naphthylmethyl Addition reactions of 1-naphthylmethyl with radicals and oxidation of the products	$A_{2}ĊH_{2}+\;\dot{O}H$$=A_{2}{\mathrm{CH}}_{2}\dot{O}$ + Ḣ	1.00 × 10^13^	0.5	0	0	Narayanaswamy [14]
	$A_{2}ĊH_{2}+Ö=A_{2}{\mathrm{CH}}_{2}\dot{O}$	1.14 × 10^14^	0.5	0	0	Narayanaswamy [14]
	$A_{2}ĊH_{2}+O_{2}=A_{2}\mathrm{CHO}+\;\dot{O}H$	2.00 × 10^13^	2.0	0	40,000	Creck [15]
	$A_{2}ĊH_{2}+\;\dot{O}H$ = A_2_CH_2_OH	1.00 × 10^13^	0.5	0	0	Wang [11]
Addition reactions of 1-naphthylmethyl with radicals and oxidation of the products Decomposition of 1-naphthylmethyl-oxy	A_2_ĊH_2_ + ĊH_3_ = A_2_C_2_H_5_	5.95 × 10^12^	0.5	0	221	Wang [11]
	$A_{2}{\mathrm{CH}}_{2}\mathrm{OH}+O_{2}=>\;A_{2}\mathrm{CHO}+{H\dot{O}}_{2}$ + Ḣ	6.00 × 10^14^	2.0	0	41,348	Wang [11]
	$A_{2}{\mathrm{CH}}_{2}\mathrm{OH}+O_{2}=A_{2}{\mathrm{CH}}_{2}\dot{O}$ $+{H\dot{O}}_{2}$	1.00 × 10^14^	0.5	0	41,400	Wang [11]
	$A_{2}{\mathrm{CH}}_{2}\dot{O}$ = A_2_CHO + Ḣ	2.63 × 10^28^	2.0	−5.08	22,249	Narayanaswamy [14]
Oxidation of naphthalene	A_2_ + Ö = A_2_OH	3.66 × 10^13^	2.0	0	4529	Narayanaswamy [14]
Decomposition of indene	Ċ_9_H_7_ + Ḣ = C_9_H_8_	6.32 × 10^13^	0.5	0.281	179	NUIG-mech [24]
Oxidation of indenyl	${Ċ}_{9}H_{7}+{H\dot{O}}_{2}$ $=C_{9}H_{7}\dot{O}$ $+\;\dot{O}H$	7.50 × 10^12^	0.5	0	0	Wang [11]

## Data Availability

Data are contained within the article and Supplementary Materials.

## Supplementary Materials

The following supporting information can be downloaded at: https://www.mdpi.com/article/10.3390/molecules29235660/s1.
